# Anthropometric Indices of Obesity and the Prediction of Cardiovascular Risk Factors in an Iranian Population

**DOI:** 10.1100/tsw.2009.58

**Published:** 2009-06-12

**Authors:** Mohsen Azimi–Nezhad, Majid Ghayour–Mobarhan, Mohammad Safarian, Habibollah Esmailee, Seyed Mohammad Reza Parizadeh, Maryam Rajabi-Moghadam, Arezoo Gholami, Mohammad Reza Oladi, Gordon A. Ferns

**Affiliations:** ^1^ Department of Biochemistry and Nutrition, Mashhad University of Medical Sciences (MUMS), Mashhad, Islamic Republic of Iran; ^2^ Cardiovascular Research Center, Avicenna Research Institute, Mashhad University of Medical Sciences (MUMS), Mashhad, Islamic Republic of Iran; ^3^ Community Health and Statistic Department, Mashhad University of Medical Sciences (MUMS), Mashhad, Islamic Republic of Iran; ^4^ Pharmacutical Research Center, Avicenna Research Institute, Faculty of Medicine, Mashhad University of Medical Sciences (MUMS), Mashhad, Islamic Republic of Iran; ^5^ Postgraduate Medical School, University of Surrey, Stag Hill, Uk

**Keywords:** anthropometric, cardiovascular risk, Iran, ROC curve

## Abstract

The prevalence of hypertension, diabetes, dyslipidemia, and metabolic syndrome are increasing globally. The present study was conducted in an attempt to define optimal cutoff values for several anthropometric variables in an Iranian population, as these may vary with ethnicity. Iranian subjects (2483 men and 2445 women), aged 15–65 years, were recruited using a cluster-stratified sampling method from rural and urban areas within the Khorasan province. Receiver operating characteristics (ROC) analysis was used to define optimal anthropometric cutoff values. The prevalence of hypertension, diabetes, dyslipidemia, and metabolic syndrome were 28, 5.5, 67, and 39.9%, respectively. The gender-specific cutoff values for waist:height ratio to predict hypertension, diabetes, dyslipidemia, and metabolic syndrome among men were 0.52 (sensitivity = 66%; specificity = 66%), 0.54 (sensitivity = 65%; specificity = 65%), 0.50 (sensitivity = 58%; specificity = 57%), and 0.53 (sensitivity = 73%; specificity = 70%), and for women were 0.59 (sensitivity = 61%; specificity = 61%), 0.61 (sensitivity = 64%; specificity = 64%), 0.57 (sensitivity = 61%; specificity = 61%), and 0.59 (sensitivity = 77%; specificity = 77%) (*p* < 0.05). Significant correlations were found between waist:height ratio and hypertension, diabetes mellitus, dyslipidemia, and metabolic syndrome, particularly in women. Waist circumference cutoffs were higher for women than men for hypertension, diabetes mellitus, and dyslipidemia.

## INTRODUCTION

Cardiovascular disease is common and has imposed a considerable burden on the Iranian health system[1,2]. Overweight and obesity have been found to be major determinants of chronic diseases that include diabetes, hypertension, and metabolic syndrome, and these are growing in prevalence in both developed and developing countries[3,4].

In prospective studies, indices of obesity, such as body mass index (BMI), waist circumference (WC), waist:hip ratio (WHR), and waist:height ratio (WHtR), have been associated with an increased risk of hypertension (HTN)[5], type II diabetes mellitus (DM), and metabolic syndrome (Met.S) regardless of age and ethnicity[4,6]. BMI is generally accepted as a good estimate of general obesity, while other indices are indicators of central adiposity[7]. A good understanding of the relationships between anthropometric indices and chronic diseases, such as DM, HTN, and Met.S, has important clinical and public health implications. It can, for example, help to refine appropriate anthropometry reference ranges for obesity and its complications. It may also be possible to use these indices to guide the targeting of resources in the prevention and management of obesity.

However, cutoff values for these anthropometric indices may vary between populations. In South Asians, for example, the relationship between risk of DM and BMI appears to differ from Caucasian populations[8,9].

This study was, therefore, conducted to define the best anthropometric indices and their cutoff values to identify subjects at highest risk for DM, HTN, dyslipidemia, and Met.S in an Iranian adult population.

## MATERIAL AND METHODS

### Subjects

A total of 4928 individuals (2483 men and 2445 women), aged 15–65 years, were selected using a cluster-stratified method from rural and urban areas in Khorasan Province in northeastern Iran, as part of a national cross-sectional study conducted by the Ministry of Health and Medical Education in 2004. From eligible addresses, 250 clusters were randomly selected proportional to the number of households within each region, using the probabilities required to produce the target sample size of 5000, with only one person being selected per household. Subjects were assessed for food intake, blood status indices, and anthropometric and demographic measures. Clinical examinations were carried out by trained nurses and general practitioners, joined together as research team.

### Anthropometric Measurements

Body weight was measured using a calibrated counterweight balance (Seca, Japan), personal scales to the nearest 100 g, on a hard level surface, while the subjects were wearing light clothing and were asked to remove shoes, heavy outer garments, heavy jewelry, and loose change and keys. Height was also measured at the same time with a portable, telescopic stadiometer to the nearest millimeter, while the participant's head was in the Frankfurt plane. BMI was calculated as weight (kg)/height ($m^2$). WC was measured at the midpoint between the highest point of the iliac crest and the lowest part of the costal margin. WHtR was calculated as WC (cm)/height (cm).

### Blood Pressure Measurement

Blood pressure was measured using a mercury sphygmomanometer with suitable cuff size for each subject on two occasions, separated by an interval of 15 min, while subjects were in a sitting position. The average of the two measurements of Korotkoff phase I was considered as systolic blood pressure (SBP) and the average of two values of phase IV were recorded for diastolic blood pressure (DBP).

### Biochemical Measurements

Blood samples were obtained in the early morning after an overnight fast, at the subject's home, using heparinized tubes. Samples were assayed for plasma glucose, total cholesterol, low-density lipoprotein cholesterol (LDL-C), and high-density lipoprotein cholesterol (HDL-C) using standard techniques on a Cobas autoanalyzer system.

### Definitions

According to the WHO definition, a BMI of 25–29.99 and ≥30 kg/m$^2$ were taken as cutoff values defining overweight and obesity, respectively. Based on the ATP III definitions, central obesity was defined as WC >102 cm for males and >88 for females[10].

Subjects were defined as having DM if they had a fasting plasma glucose ≥126 mg/dl (≥7 mmol/l) or where there was documented evidence of DM in their medical records, or treatment with hypoglycemic agents.

HTN was defined as a SBP ≥140 mmHg and a DBP ≥90 mmHg according to the U.S. Sixth Joint National Committee on Detection, Evaluation and Treatment of Hypertension (JNC VI) criteria[14].

Dyslipidemia was defined as a total cholesterol (TC) ≥5.2 mmol/l (200 mg/dl), triglycerides (TG) ≥1.5 mmol/l (150 mg/dl), and HDL-C <0.9 mmol/l (40 mg/dl) (for men), and <1.29 mmol/l (50 mg/dl)(for women), corresponding to the ATP III criteria for borderline high TC, TG, and low HDL-C levels[10].

Met.S was defined using ATP III criteria: the presence of three or more of the following criteria[10]:

1. WC >102 and >88 cm for men and women, respectively
2. Fasting blood glucose >110 mg/dl
3. Fasting blood TG >150 mg/dl
4. SBP and DBP >130 and >90 mmHg, respectively
5. HDL <50 and <40 mg/dl for men and women, respectively

### Data Analysis

Data were analyzed using the Statistical Package for Social Sciences (SPSS) 11.5 (SPSS Inc., Chicago), with descriptive statistics (mean and standard deviation [mean ± SD]) measured by one-way ANOVA for normal distributed data, nonparametric tests (Mann Whitney) for non-normal distributed data, and ANCOVA for adjusting important confounding factors including age and gender.

Receiver operating characteristic (ROC) curves were used to compare the various anthropometric measures to predict the presence of HTN, DM, Met.S, and dyslipidemia[11]. ROC curves were constructed for WC, BMI, and WHtR, with respect to their ability to predict the presence of the following risk factors: DM, HTN, dyslipidemia, and Met.S. The optimal cutoff point for each anthropometric measure for men and women was determined as the point of convergence of sensitivity and specificity. This was completed for men and women separately, by 10-year age groups, and for all ages combined. Pearson correlation was used to determine the relationship between anthropometric indices and individual cardiovascular disease risk factors.

### RESULTS

The characteristics of the subject groups according to sex and age are presented in Table 1. The overall prevalence of obesity was 7.8% in men and 19.7% in women ($p < 0.001$). Central obesity was found in 11.7% of men and 51.3% of women ($p < 0.001$).

**TABLE 1**
**Mean ± SD of WC, BMI, and WHtR by Sex and Age Groups**

| | Age Groups (year) | No. | WC (cm) | BMI (kg/m2) | WHtR |
|---|---|---|---|---|---|
| Men | <20 | 280 | 74.63 ± 12.54 | 20.64±3.45 | 0.43±0.07 |
| | 20–29 | 476 | 81.54 ± 11.46 | 22.84 ± 3.98 | 0.47 ± 0.06 |
| | 30–39 | 477 | 84.77 ± 12.75 | 23.77 ± 3.83 | 0.50 ± 0.07 |
| | 40–49 | 554 | 88.45 ± 13.10 | 24.78 ± 4.12 | 0.52 ± 0.07 |
| | 50–59 | 414 | 90.74 ± 12.62 | 24.98 ± 4046 | 0.54 ± 0.07 |
| | >60 | 282 | 9062 ± 12.6.2 | 25.0.3 ± 4.10 | 0.54 ± 0.07 |
| | All | 2483 | 85.49 ± 13.66 | 23.81 ± 4.25 | 0.50 ± 0.08 |
| Women | <20 | 260 | 75.09 ± 10.6 | 21.47 ± 3.34 | 0.47 ± 0.06 |
| | 20–24 | 469 | 81.24 ± 14.77 | 23.44 ± 4.33 | 0.51 ± 0.09 |
| | 30–39 | 465 | 88.19 ± 14.91 | 26.07 ± 5.40 | 0.56 ± 0.09 |
| | 40–49 | 525 | 92.82 ± 15.52 | 27.20 ± 5.30 | 0.62 ± 0.09 |
| | 50–59 | 467 | 95.13 ± 14.45 | 27.21 ± 5.53 | 0.62 ± 0.09 |
| | >60 | 258 | 94.97 ± 14.03 | 26.29 ± 5.58 | 0.622 ± 0.09 |
| | All | 2445 | 88.49 ± 15.65 | 25.44 ± 5044 | 0.57 ± 0.10 |

Values express as mean ± SD, one-way ANOVA test was used for determining of differences between groups, and $p < 0.001$ for all age categories and both genders.

The prevalence of HTN in urban and rural areas was 28.8 and 26.5% ($p = 0.006$), for DM 7 and 3% ($p < 0.001$), for Met.S (according to ATP III definition) 44.4 and 33.1% ($p < 0.001$), and for dyslipidemia 65.7 and 69.3 ($p = 0.01$), respectively (Table 2). As we have reported previously, the prevalence of DM in people under the age of 20 years is notable[12].

### Cutoff Points

Cutoff thresholds for WC, BMI, and WHtR, where sensitivity approximated to specificity for each risk factor (all ages combined) are shown in Table 3.

For the individual cardiovascular disease risk factors in men, there were different cutoff thresholds for WC between 85 to 95 cm with 85% sensitivity and 71% specificity; and for women between 89 to 96 cm with 70% sensitivity and 65% specificity.

**TABLE 2**
**Prevalence (%) of Multiple Risk Factors in Different Gender and Age Groups**

| | | <20 | 20–29 | 30–39 | 40–49 | 50–59 | >60 | All |
|---|---|---|---|---|---|---|---|---|
| Men | Met.S | 0 | 16.9 | 23.2 | 26.6 | 39.2 | 39.6 | 28.8 |
| | Dyslipidemia | 39.3 | 63.5 | 61.9 | 71.3 | 70.2 | 69 | 67 |
| | HTN | 2.9 | 4.4 | 8 | 13.9 | 25.8 | 33 | 13.8 |
| | DM | 9.5 | 0.8 | 2.5 | 5.1 | 6.8 | 10.6 | 5.1 |
| Women | Met.S | 0 | 14.7 | 33.5 | 53.9 | 65 | 76 | 49.9 |
| | Dyslipidemia | 44.4 | 51.3 | 60.1 | 66.5 | 79 | 80.8 | 67.1 |
| | HTN | 1.1 | 2 | 4.8 | 15.8 | 31.7 | 40.7 | 14.9 |
| | DM | 4.8 | 1.5 | 1.3 | 6 | 9.9 | 11.3 | 5.8 |

**TABLE 3**
**Area under the ROC Curves for Anthropometric Measures and Cardiovascular Disease Risk Factors**

| | Men | | | Women | | |
|---|---|---|---|---|---|---|
| | WC | BMI | WHtR | WC | BMI | WHtR |
| Met.S | 0.79 | 0.75 | 0.78 | 0.84 | 0.76 | 0.83 |
| Dyslipidemia | 0.58 | 0.59 | 0.60 | 0.62 | 0.61 | 0.63 |
| HTN | 0.70 | 0.69 | 0.71 | 0.71 | 0.66 | 0.71 |
| DM | 0.69 | 0.62 | 0.70 | 0.69 | 0.66 | 0.70 |

**TABLE 4**
**Cutoff Values for Anthropometric Indices where Sensitivity Approximated to Specificity when Predicting Individual and Multiple Risk Factors for Men and Women**

| | Men | | | Women | | |
|---|---|---|---|---|---|---|
| | WC (cm) | BMI (kg/m$^2$) | WHtR | WC (cm) | BMI (kg/m$^2$) | WHtR |
| Met.S | 90 | 25 | 0.53 | 90 | 25.77 | 0.59 |
| Dyslipidemia | 85 | 24 | 0.50 | 89 | 25.42 | 0.57 |
| HTN | 87 | 24.6 | 0.52 | 92 | 26 | 0.59 |
| DM | 92 | 24.96 | 0.54 | 96 | 27 | 0.61 |

For BMI, there was a limited variation of cutoff values compared to WC for both genders (Table 4). However, there was a small range of cutoff values for WHtR indices in men and women. The differences of WHtR for men and women varied between 0.50 and 0.54, and 0.57 and 0.63, respectively (Table 3).

### Differences of Areas under ROC Curve

The areas under ROC curve for WHtR for men ranged from 0.49 to 0.85, and for women between 0.51 and 0.83 ($p < 0.001$). The values obtained for BMI in men ranged between 0.49 and 0.79, and for women between 0.51 and 0.76. This statistical variable was calculated for WC between 0.49 and 0.83, and 0.50 and 0.84, for men and women, respectively ($p < 0.001$, Table 3).

The overall performance of the ROC curve was used to determine the optimal cutoff values for the population. The optimal cutoff values for WC, BMI, and WHtR were 93.75 cm (sensitivity = 71%; specificity = 70%), 26.30 (sensitivity = 69%; specificity = 68%), and 0.58 (sensitivity = 73%; specificity = 70%), respectively. The corresponding areas under the ROC curve were 0.77, 0.73, and 0.78.

Pearson correlation test showed that the WHtR was most strongly associated with the presence of Met.S (r = 0.51), dyslipidemia (r = 0.15), HTN (r = 0.25), and DM (r = 0.15). The correlations for BMI were 0.45, 0.15, 0.21, and 0.12 for Met.S, dyslipidemia, HTN, and DM, respectively. The respective relationships for WC were less strong than for the WHtR.

### DISCUSSION

This study has attempted to define the optimum cutoff values for the anthropometric parameters WC, BMI, and WHtR for the prediction of several important cardiovascular risk factors. The optimum cutoff values were derived primarily by analysis of sensitivity and specificity values. Our results indicate that ranges of cutoff values for each of these anthropometric variables may be considerable. Optimal cutoff values are dependent on sex, age, and the prevalence of the risk factor being screened. The values for the cutoffs were found to be higher among women, which differs from some other studies[13]. Higher cutoff values were also found to be more appropriate for older age groups.

We found there was little variation in the optimal cutoff values for WHtR, which were 0.5 for men and 0.6 for women. These values are similar to those previously reported by others[2,14] and are consistent with previous studies in concluding that the WHtR is probably the best single anthropometric measure to use in identifying individuals with risk factors for cardiovascular disease[15].

Although some investigators have proposed that WC is a superior indicator because it requires only one measurement and correlates well with visceral adiposity[16], our results indicate that WHtR is better than WC in our population, and for practical reasons, the values of 0.5 for men and 0.6 for women may be the most pragmatic measures to use for the population at large, as they are memorable. This finding is consistent with other reports from studies in Japan and India[17,18].

In the present study, the WHtR was the strongest predictor of HTN and Met.S. There were no significant differences in the correlation between other anthropometric indices with BMI and DM.

For BMI, the optimal cutoff values ranged between 25.4 and 27.8 for women, and 25 and 26 for men. The global standard of BMI ≥ 25 for measurement of overweight for both sexes falls in both of these ranges[8].

Optimal values of WC fall into a wider range (85–95 cm for men and 89–94.5 cm for women) and are dependent on the cardiovascular risk factor being considered.

In some previous studies, the WHtR in men and WC in women were reported to be the strongest predictors of the cardiovascular disease risk factors, but reports are inconsistent[19].

We have found that WHtR was the best screening measure for cardiovascular risk factors, when compared with BMI, and WC, which is consistent with the study of Hseih et al. that showed that the WHtR was more highly correlated with several cardiovascular disease risk factors than WC[10].

The results of other studies in Iran have suggested that the cutoff values of anthropometric measures as indicators of cardiovascular risk factors are higher for Iranians than other Asian populations[2].

The principal limitation of this current study has been the use of cross-sectional data to identify the cutoff values for the anthropometric indices, rather than prospective data. In this respect, one of the related indices, the WHR index, was not available for comparison in this paper. We suggest that future studies using a prospective design could be used to assess the validity of these cutoff points.

### ACKNOWLEDGMENT

We would like to thank Mashhad University of Medical Sciences for financial support of this study.
